# Transcriptional Responses of *Herbaspirillum seropedicae* to Environmental Phosphate Concentration

**DOI:** 10.3389/fmicb.2021.666277

**Published:** 2021-06-10

**Authors:** Mariana Grillo-Puertas, Josefina M. Villegas, Vânia C. S. Pankievicz, Michelle Z. Tadra-Sfeir, Francisco J. Teles Mota, Elvira M. Hebert, Liziane Brusamarello-Santos, Raul O. Pedraza, Fabio O. Pedrosa, Viviana A. Rapisarda, Emanuel M. Souza

**Affiliations:** ^1^Instituto de Química Biológica, “Dr. Bernabé Bloj”, Facultad de Bioquímica, Química y Farmacia, Universidad Nacional de Tucumán (UNT) and Instituto Superior de Investigaciones Biológicas (INSIBIO), CONICET-UNT, San Miguel de Tucumán, Argentina; ^2^Department of Biochemistry and Molecular Biology, Universidade Federal do Paraná, Curitiba, Brazil; ^3^Centro de Referencia para Lactobacilos (CERELA-CONICET), San Miguel de Tucumán, Argentina; ^4^Facultad de Agronomía y Zootecnia, Universidad Nacional de Tucumán (UNT), San Miguel de Tucumán, Argentina

**Keywords:** *Herbaspirillum seropedicae*, polyphosphate, adhesion, phosphate, plant-microbe interaction

## Abstract

*Herbaspirillum seropedicae* is a nitrogen-fixing endophytic bacterium associated with important cereal crops, which promotes plant growth, increasing their productivity. The understanding of the physiological responses of this bacterium to different concentrations of prevailing nutrients as phosphate (Pi) is scarce. In some bacteria, culture media Pi concentration modulates the levels of intracellular polyphosphate (polyP), modifying their cellular fitness. Here, global changes of *H. seropedicae* SmR1 were evaluated in response to environmental Pi concentrations, based on differential intracellular polyP levels. Cells grown in high-Pi medium (50 mM) maintained high polyP levels in stationary phase, while those grown in sufficient Pi medium (5 mM) degraded it. Through a RNA-seq approach, comparison of transcriptional profiles of *H. seropedicae* cultures revealed that 670 genes were differentially expressed between both Pi growth conditions, with 57% repressed and 43% induced in the high Pi condition. Molecular and physiological analyses revealed that aspects related to Pi metabolism, biosynthesis of flagella and chemotaxis, energy production, and polyhydroxybutyrate metabolism were induced in the high-Pi condition, while those involved in adhesion and stress response were repressed. The present study demonstrated that variations in environmental Pi concentration affect *H. seropedicae* traits related to survival and other important physiological characteristics. Since environmental conditions can influence the effectiveness of the plant growth-promoting bacteria, enhancement of bacterial robustness to withstand different stressful situations is an interesting challenge. The obtained data could serve not only to understand the bacterial behavior in respect to changes in rhizospheric Pi gradients but also as a base to design strategies to improve different bacterial features focusing on biotechnological and/or agricultural purposes.

## Introduction

Beneficial plant–bacteria interactions promote plant growth and development. Plant growth-promoting bacteria (PGPB) represent a promising alternative to be used as biofertilizer and biocontrol agent, due to their ability to fix atmospheric N_2_, solubilize minerals, and produce phytohormones and siderophores (Bhattacharyya and Jha, 2012). Environmental conditions before, during, and after plant inoculation can influence the effectiveness of the PGPB (Berney et al., 2006; Baldani et al., 2014). It is well known that bacteria are influenced by the soil conditions including temperature, moisture, and chemicals (Glick et al., 1999). Data regarding concentration of phosphorus in the soil solution are scarce and inconsistent. An adequate phosphate (Pi) supply is a challenge for many bacteria in soil, where the level of free Pi can be affected by the pH-dependent formation of precipitates (Bagyaraj et al., 2000; Matula, 2011). Pi concentration in soil produces changes in microbial community composition (Leff et al., 2015; Fabiañska et al., 2018). In addition, Pi availability influences the plant–microbe interaction along the mutualism process (Hiruma et al., 2016).

Pi is an essential constituent of all living organisms. Biomolecules containing Pi participate in a wide range of cellular activities, including processing of cellular information, energy metabolism, signaling, regulation of protein activity, and maintenance of acid–base homeostasis (Devine, 2018). In bacteria, the mechanism that senses and responds to Pi limitation involves the high-affinity phosphate-specific transport (Pst) system, in combination with the two-component signaling system, PhoR–PhoB, collectively known as the phosphate regulon (Pho regulon) (Hsieh and Wanner, 2010). The most common genes of the Pho regulon encode extracellular enzymes capable of obtaining Pi from organic phosphates, Pi-specific transporters, and enzymes involved in nutrient storage processes (Rao and Torriani, 1990; Santos-Beneit et al., 2008; Hsieh and Wanner, 2010). The Pho regulon not only is a regulatory circuit of Pi homeostasis but also plays an important adaptive role in bacterial stress and virulence (Chekabab et al., 2014).

Microorganisms store Pi in the form of polyphosphate (polyP), a linear polymer of Pi with a chain length of 1,000 residues or more. Besides its function as a storage compound, polyP is involved in stress response (e.g., starvation, acid, or oxidative stresses) virulence, motility, or biofilm formation, among other physiological processes (Grillo-Puertas et al., 2012, 2014, 2015, 2018; Gray and Jakob, 2015; Albi and Serrano, 2016). It has been described in several bacteria that variations in environmental Pi concentrations can modulate intracellular polyP levels, affecting bacterial physiology (Schurig-Briccio et al., 2009a; Grillo-Puertas et al., 2012, 2014, 2015, 2016, 2018; Correa-Deza et al., 2017). Indeed, *Escherichia coli* K-12 cells maintained a high polyP level during the stationary phase in the presence of a high Pi concentration (>25–37 mM). High polyP levels were correlated to protection against oxidative stress, inhibition of biofilm formation, and tolerance to copper salts (Schurig-Briccio et al., 2009b; Grillo-Puertas et al., 2012, 2015). In the PGPB *Gluconacetobacter diazotrophicus*, variations in media Pi concentration modulated the intracellular polyP levels, affecting its survival, environmental stress tolerance, biofilm formation capacity, and competence as a promoter of strawberry plant growth (Grillo-Puertas et al., 2018).

*Herbaspirillum seropedicae* is a diazotrophic endophytic PGPB that colonizes several crops, such as rice (*Oryza sativa*), maize (*Zea mays*), sorghum (*Sorghum bicolor*), and sugarcane (*Saccharum officinarum*). Within the *H. seropedicae* genome, four genes coding for polyP-related proteins were found: *Hsero_1254* (*ppk1*), *Hsero_3790* (*ppk2*), and *Hsero_4359*, encoding enzymes involved in polyP synthesis, and *Hsero_1891* (*ppx*), coding for an exopolyphosphatase (Pedrosa et al., 2011). Here, the global changes of *H. seropedicae* SmR1 in response to environmental Pi conditions, based on differential intracellular polyP levels, were evaluated through RNA-seq along with physiological experiments. Our results provide valuable insights into the *H. seropedicae* gene expression upon differential Pi conditions, and allowed to assess its role in several adaptive pathways.

## Materials and Methods

### Bacterial Strain, Culture Media, and Growth Conditions

*H. seropedicae* strain SmR1 (Souza et al., 2000) was routinely grown at 30°C with 120 rpm in NFbHPN-malate medium (Pedrosa and Yates, 1984; Klassen et al., 1997) supplemented with 80 μg ml^–1^ of streptomycin. NFbHPN containing 50 mM potassium phosphate buffer was denoted here as NFb50 medium. Also, NFbHPN-malate was prepared with a reduced Pi concentration of 5 mM and defined as NFb5 medium. Growth curves were performed in both media at 30°C for 24 h. Aliquots were taken at different time intervals to measure CFU ml^–1^ and absorbance at 600 nm.

### Measurement of PolyP Levels

Intracellular polyP was measured in cell suspensions using a DAPI (4’,6-diamidino-2-phenylindole)-based fluorescence approach (Aschar-Sobbi et al., 2008). Briefly, cells were grown in NFb5 or NFb50 media at 30°C for 24 h. Cell suspensions were washed and resuspended in buffer T (100 mM Tris–HCl, pH 7.5) at an A_600 nm_ = 0.02. For cell permeabilization, 15 μl of 0.1% SDS and chloroform were added. Finally, 17 μM DAPI (Sigma) was added to cuvettes. After stirring for 5 min at 37°C, the DAPI fluorescence spectra (excitation, 415 nm; emission, from 445 to 650 nm) were recorded using an ISS PCI spectrofluorometer (SS Inc., Champaign, IL). Fluorescence (expressed as arbitrary units) of the DAPI–polyP complex at 550 nm was used as a measure of the intracellular polyP level, since fluorescence emissions from free DAPI and DAPI-DNA are minimal at this wavelength (Aschar-Sobbi et al., 2008).

### Transcriptome Profiling RNA-Seq Design and Analyses

*H. seropedicae* SmR1 cells were grown in NFb5 or NFb50 media and harvested after 9 h of growth with a cell density corresponding to an A_600 nm_ ∼ 0.8 for both conditions. Three independent samples (biological replicates) of each condition were used to construct six sequencing libraries. Bacterial samples were taken, and total RNA was isolated using TRI Reagent (Sigma) followed by chloroform extraction as recommended by the manufacturer. Samples were treated with DNaseI (Ambion) and the RNA was quantified with a NanoDrop Spectrophotometer (THERMO Scientific) and gel electrophoresis. The ribosomal RNA was depleted using Ribo-Zero rRNA Removal Kit-Gram-Negative Bacteria (Illumina). The whole transcriptome libraries were constructed using the Ion total RNA-seq Kit v2 (Thermo Fisher Scientific), barcode with Ion Xpress^TM^ Barcode Adapters, following the manufacturer instructions. The libraries were amplified and enriched using the Ion PI Hi-QTM OT2 200 Kit, and sequenced with the Ion PI Hi-QTM Sequencing 200 kit on an Ion PITM Chip Kit v3. Obtained sequences were trimmed, mapped, and analyzed using CLC Genomics Workbench 7.5.1 (Qiagen) against the *H. seropedicae* SmR1 genome (NC_014323). The following parameters were used: reads were trimmed to a minimum of 40 bp, 90% alignment to the reference sequence and 80% identity required for inclusion as a mapped read, number of hits equal to 1, and number of additional bases downstream and upstream of the CDS equal to 50 bp. A gene was considered to be expressed with a read coverage equal to or higher than threefold and differentially expressed when the normalized RPKM (reads per kilobase per million mapped reads) value was twofold higher in NFb50 compared to NFb5 medium and with a *p* ≤ 0.05. Differential gene expression was accessed using the DESeq2 package, which is based on a negative binomial distribution analysis (Love et al., 2014). Those genes with a RPKM log_2_fold change ≥ 1 and *p* ≤ 0.05 were considered differentially regulated. Some genes with fold changes marginally lower than 2.0-fold were also considered regulated, if neighborhood analysis suggested that they are part of a pathway with genes regulated according to the previous criteria. Differential expression values in the text, tables, and figures are shown as fold change.

### Quantitative Reverse Transcription Real-Time PCR

In order to confirm differential expression in the RNA-seq profiling, quantitative reverse transcription real-time PCR was performed on selected targets. Briefly, 2 μg of RNA extracted from *H. seropedicae* as described previously was used to synthesize cDNA using High Capacity cDNA Reverse Transcription Kit (Applied Biosystems) and quantified in triplicate using the Power SYBR-Green PCR MasterMix on a Step-One Plus Real Time-PCR System (Applied Biosystems). The PrimerQuest Tool of Integrated DNA Technologies^®^ was used to design qPCR primers, as listed in Table 1. Primer efficiency was calculated through cDNA dilution curve over at least five orders of magnitude. The 16S rRNA gene was used as internal control, and the relative gene expression was determined using the 2^–ΔΔCt^ method (Livak and Schmittgen, 2001).

**TABLE 1:** Primers used for qPCR.

Primers	Sequence 5′–3′	Target	Reference
FlhA-f	GAAATTCGCCCAGGAAGT	*flhA*	This work
FlhA-r	CCCTTCAGAGTAATGCGATAG	*flhA*	This work
CheD-f	CTTCGCATCCTACGTCTATC	*cheD*	This work
CheD-r	CTCGCCGGTATAGAAATACTC	*cheD*	This work
RfbD-f	CGATGAAGAGTTCCGGATTG	*rfbD*	This work
RfbD-r	GTCTTCGTACATTGCCTCTATC	*rfbD*	This work
16S-f	AAGCCTACCAAGGCGACGACGAT	*16S*	This work
16S-r	AGGAGTCTGGGCCGTGTCT	*16S*	This work

**Primers were designed using The PrimerQuest Tool of Integrated DNA Technologies^®^.**

### Determination of Alkaline Phosphatase Activity

Alkaline phosphatase (AP) is an enzyme encoded by *phoA*, a Pho regulon member in several bacteria (Wanner and Latterell, 1980; Lamarche et al., 2005, 2008). AP activity was determined using the chromogenic substrate *p*-nitrophenylphosphate (pNPP), according to a previously described method (Lamarche et al., 2005) with modifications. Briefly, cells were grown at 30°C in the indicated media and aliquots were extracted at different times. For permeabilization, cells were resuspended at an A_600 nm_ = 0.5 in 1 M Tris–HCl buffer (pH 8) to a final volume of 1 ml with the addition of 30 μl of 0.1% SDS and 30 μl of chloroform and incubated for 30 min at room temperature. Permeabilized cells were incubated with 2 mM pNPP (Sigma) at 37°C for 20 min or up to color development. Absorbance at 405 and 550 nm was determined. AP activity was calculated with the following equation, where *t* is the reaction time in minutes:

$$\text{AP}\;\text{activity}\;=\;{\text{A}}_{450\text{nm}}/(\text{t}\;\times \;{\text{A}}_{550\text{nm}}).$$

### Pi Solubilization Assay

For Pi solubilization assay, cells were first grown in M1 liquid medium (Gupta et al., 1999). After 24 h of incubation at 30°C and 150 rpm, cells were harvested by centrifugation (10,000 rpm; 5 min). The pellet was washed twice and resuspended in 0.9% NaCl solution (w/v) to an A_600 nm_ = 0.3. Aliquots of 10 μl were placed in NBRIP plates containing 5 g L^–1^ tricalcium phosphate [Ca_3_(PO_4_)_2_] (Nautiyal, 1999), and NBRIP was supplemented with 5 or 50 mM Pi buffer (denoted as NBRIP+5 or NBRIP+50, respectively). After incubation at 30°C for 72 h, the solubilization index (SI) was determined as previously described by Edi–Premono et al. (1996). A positive reaction was determined as the presence of clear halos around the colonies.

### Motility and Chemotaxis Assays

Overnight NFb50 cultures were washed and resuspended in fresh NFb5 medium to an A_600 nm_ = 0.1. Motility was evaluated according to Ulett et al. (2006) with modifications. A cell suspension was spotted onto the center of a NFb5 or NFb50 0.3% agar plates, using a sterile toothpick for inoculation. Plates were incubated at 30°C for 48 h, and swimming motility was determined by measuring diameters of growth. Data are expressed in centimeters as the mean diameter of movement for four independent experiments. Chemotaxis was evaluated by measuring the distance of the bacterial growth from the spot to the edge of the halo in the direction of chemical stimuli (malate, glucose, salicylic acid, shikimic acid, and root exudates) in 0.3% agar plates containing 0.9% NaCl without a carbon source, after 48 h at 30°C. Inoculation point was 1.5 cm away from the attractants. Solutions of 4% malate or glucose and 75 μg ml^–1^ shikimic or salicylic acids were used. For root exudates, a filter disc containing exudates from germinated maize seeds were placed on the plates.

### Quantification of Biofilm Formation

Biofilm formation was assayed by growing cells on polystyrene microtiter plates and stained with crystal violet solution (O’Toole and Kolter, 1998). Overnight cultures in the NFb50 medium were diluted to an A_600 nm_ = 0.1 with fresh NFb5 or NFb50 media. Two hundred microliters of cells were grown in a 96-well microtiter plate under static conditions at 30°C for 48 h. Then, unattached cells were removed by washing the plates with deionized water. Two hundred microliters of 0.1% crystal violet solution were added to each well, and plates were incubated at room temperature for 15 min. Then, wells were rinsed three times with water. The absorbed crystal violet was extracted with 200 μl of 95% ethanol, and A_595 nm_ was measured (Spectra MaxPlus384 Absorbance Microplate Reader, United States).

### Maize Root Colonization Assay

Assays of maize (*Z. mays*) root colonization by *H. seropedicae* SmR1 strain were performed according to Balsanelli et al. (2013) with modifications. Briefly, seeds of *Z. mays* cv. 30F53 (Dow AgroScience, Argentina) were surface-sterilized as described previously (Döbereiner et al., 1995; Balsanelli et al., 2010) and pre-germinated in 1% (w/v) water-agar Petri dishes in the dark for 48 h at 30°C. The seedlings were then transferred to glass tubes (one seedling in each tube) with 5 ml of Plant Medium (Egener et al., 1999) containing a final Pi concentration of 5 or 50 mM (named as PM5 or PM50, respectively) and about 2 ml in volume of sterile perlite (Perlomeoi^®^, Imerys, Brazil) as support. Each tube was inoculated with 1 ml of *H. seropedicae* cell suspension (∼10^7^ CFU ml^–1^). The inoculated maize seedlings were incubated at 26°C with a light cycle of 14/10 h (light/dark) for 5 days. Free living, root epiphytic and endophytic bacterial populations were quantified. Epiphytic bacteria were recovered by vortexing roots for 30 s in 1 ml of 0.9% NaCl solution. Endophytic bacteria were recovered by homogenizing surface-sterilized roots with a pestle and mortar. Homogenates were serially diluted and plated on NFb50 medium containing streptomycin (80 μg ml^–1^). Colonies were counted after 2 days of incubation at 30°C and expressed as colony-forming units (CFU) per milliliter of medium or per gram of fresh root tissue.

### Poly-3-Hydroxybutyrate (PHB) Quantification by Flow Cytometry

PHB quantification was carried out by flow cytometry, according to Alves et al. (2017). Briefly, *H. seropedicae* SmR1 and Δ*phaC1*, a PHB synthesis-deficient mutant (Tirapelle et al., 2013), were cultivated in NFb5 or NFb50 media for 24 h. At different time points, 100 ml of bacterial culture (∼10^6^–10^7^ cells) was centrifuged for 1 min at 13,000 rpm. The supernatant was discarded, and the cell pellet was resuspended in 1 ml of TBAC buffer [PBS buffer containing 1 mM EDTA and 0.01% (v/v) Tween 20] and 50% of ethanol (v/v). After 1 min of incubation, samples were stained with 31 mM of Nile Red for 1 min in the dark and centrifuged 1 min at 13,000 rpm, and the supernatant was discarded. The pellet was then resuspended in 1 ml of TBAC solution and immediately analyzed in a BD Accuri C5s Flow Cytometer (BD Biosciences, United States), equipped with a 488-nm laser for fluorescence excitation. For each sample, 100,000 events were acquired. The fluorescence intensities were obtained from histograms of FL2-H 585/40 nm channel.

### Statistical Analysis

When indicated, data were subjected to analysis of variance (ANOVA) followed by Tukey’s test with Infostat Analytical Software 2020 for Windows. Differences at *p*-value 0.05 were considered significant.

## Results and Discussion

### PolyP Levels in *H. seropedicae* Growing Under Different Pi Concentrations

**FIGURE 1:**
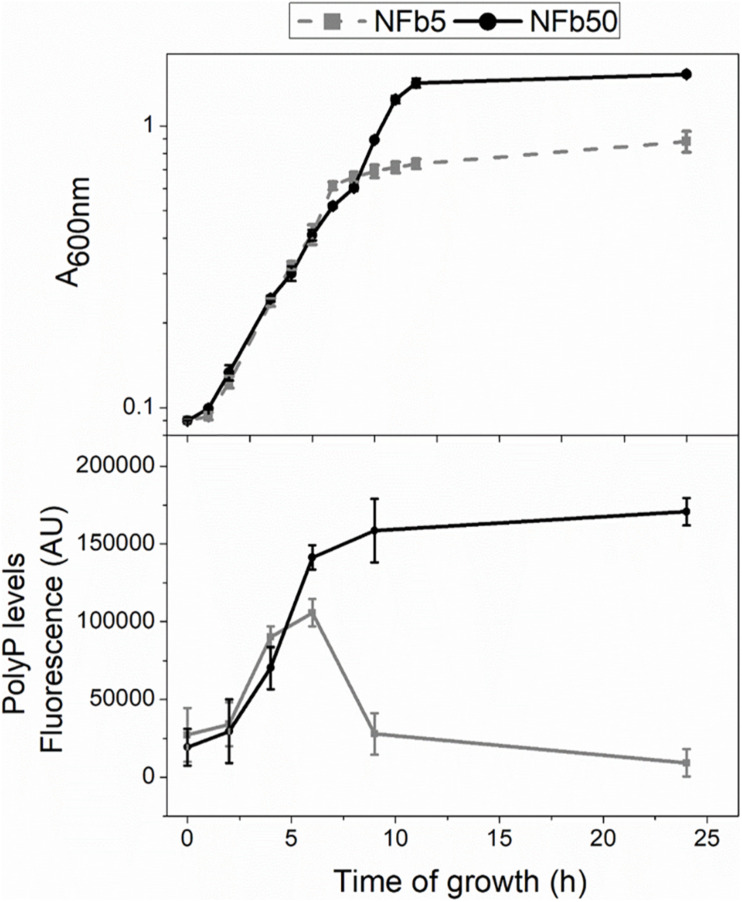
Cell growth and polyP levels in sufficient and high Pi media. Cells were grown at 30°C for 24 h in NFb5 or NFb50 media. Bacterial growth and polyP levels were measured at indicated times. Growth was determined by measuring the A_600 nm_ (upper panel). PolyP levels were determined using DAPI fluorescence and data were expressed in fluorescence arbitrary units (AU) (bottom panel). Data represent the mean ± SD of at least three independent experiments with a *p* < 0.05.

Intracellular polyP was measured in *H. seropedicae* SmR1 throughout the growth curves to establish whether media Pi concentration influences the polymer levels. Figure 1 shows growth and polyP levels in sufficient or high Pi concentration media, containing 5 or 50 mM Pi (NFb5 or NFb50, respectively). Bacterial growth was similar in both media up to early stationary phase. At 24 h, cells grown in NFb50 reached higher A_600 nm_ values than those grown in NFb5, corresponding to ∼10^8^ and 10^7^ CFU ml^–1^, respectively. SmR1 cells grown in NFb50 medium accumulated polyP up to 9 h, maintaining high levels during the stationary phase. However, cells grown in NFb5 medium accumulated the polymer up to 6 h and, thereafter, degraded it. Thus, an improved stationary phase survival of *H. seropedicae* cells was observed in NFb50, where high polyP levels were maintained. Fluctuation of polyP levels by environmental Pi has been previously reported in *E. coli* K12, uropathogenic *E. coli*, *Lactobacillus rhamnosus*, and *G. diazotrophicus* (Schurig-Briccio et al., 2009a; Grillo-Puertas et al., 2015, 2018; Correa-Deza et al., 2017). A common feature among these bacteria is the improvement of their stationary phase fitness when cells present high intracellular polyP levels, which can be achieved adjusting media Pi concentration, according to the studied microorganism.

### RNA-Seq of *H. seropedicae* Grown Under Differential Pi Conditions

Considering that environmental Pi concentration modulates polyP levels in *H seropedicae* SmR1 and to analyze the global changes generated by these variations, a comparative transcriptomic analysis by RNA-seq was carried out in cells grown in NFb5 and NFb50 media at 9 h. The selection of this particular time was based on the facts that OD and CFU (∼10^7^ CFU ml^–1^ for both cultures) values were similar at 9 h and polyP levels were remarkably different when comparing both conditions (Figure 1). After sequencing, 26 and 35 total million reads were obtained for NFb50 and NFb5 conditions, respectively, and from those, 12 million and 16 million reads were uniquely mapped to the *H. seropedicae* SmR1 genome. The transcriptomic raw data presented in this study can be found in GEO repository (GSE168341)^1^. Biological replicates showed a very high level of correlation (*r*^2^ > 0.98) (Table 2); thus, all the libraries of each condition were used for further analysis. Among the 4805 genes of the *H. seropedicae* SmR1 genome, 3976 genes were expressed considering a read coverage equal to or higher than threefold. Supplementary Table 1A shows the 1330 genes whose expression levels were statistically different (*p* ≤ 0.05), from which 670 had fold changes of 2 or higher and were considered differentially expressed genes (DEGs) in NFb50 vs. NFb5 transcriptome (Supplementary Table 1B), with 385 being downregulated and 285 being upregulated. To confirm the differential expression observed by the RNA-seq data, the regulation of *flhA*, *cheD*, and *rfbD* genes was confirmed by RT-qPCR (Table 3).

**TABLE 2:** Summary of RNA-seq data.

Samples^b^	Reads in biological samples	Total reads	Reads mapped unambiguously^a^	Correlation (*R*^2^)
NFb5 1	11,822,627	35,325,305	2,921,150	0.98
NFb5 2	13,743,184		4,203,286	
NFb5 3	9,759,494		2,232,484	
NFb50 1	8,506,901	25,578,009	2,514,583	0.99
NFb50 2	6,549,535		1,803,945	
NFb50 3	10,521,573		2,342,495	

**^*a*^The reads were uniquely mapped to the H. seropedicae SmR1 genome using CLC Genomics Workbench 7.2 with 90% of minimum length and 80% of similarity. DESeq analysis was carried out to the reads mapped to the reference genome. ^*b*^The numbers refer to biological replicates.**

**TABLE 3:** Comparison of the gene expression of *H. seropedicae* SmR1 under Pi differential conditions between reverse transcription-quantitative PCR and RNA-seq analysis.

Gene	Fold change transcriptome^a^	qPCR^b^
*cheD*	3.105	15
*flhA*	2.814	10
*rfbD*	3.007	−1.9

**^*a*^Fold change in NFb50 vs. NFb5 determined by CLC Workbench 7.5.2. ^*b*^Relative expression change in NFb50 vs. NFb5 condition by qPCR.**

**FIGURE 2:**
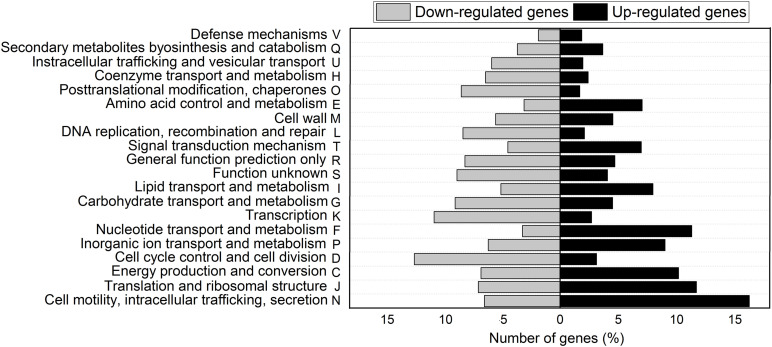
COGs classification of differentially expressed genes in NFb50 condition relative to NFb5 condition. COG functional categories are shown. Black bars indicate upregulated genes and gray bars denote downregulated genes.

Clusters of orthologous groups (COGs) classification of DEG showed that 20 biological processes were involved in the response to Pi concentration. Figure 2 shows functional classification of upregulated and downregulated genes of cells grown in Pi differential conditions. Most DEGs belong to the following functional categories: Cell motility, intracellular trafficking, and secretion (22.7%); Translation, ribosomal structure, and biogenesis (18.7%); Energy production and conversion (16.9%); Inorganic ion transport and metabolism (15%); Nucleotide transport and metabolism (14.6%); Transcription (13.5%); and Carbohydrate transport and metabolism (13.5%) (Figure 2).

A detailed knowledge-driven DEG analysis of pathways involved in relevant aspects of *H. seropedicae* SmR1 as PGPB is depicted below.

### Phosphate Metabolism

**FIGURE 3:**
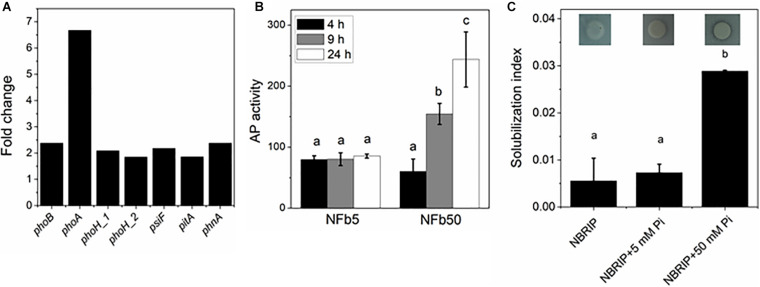
Expression of Pi metabolism-related genes, AP activity, and Pi solubilization in NFb50 condition relative to NFb5 condition. **(A)** Fold changes of differentially expressed genes. **(B)** Cells of the SmR1 strain were grown at 30°C in the indicated media. At 4, 9, and 24 h, aliquots were removed and AP activities were determined according to Materials and Methods section. **(C)** Cells of the SmR1 strain were grown at 30°C in M1 medium for 24 h and then spotted in NBRIP plates containing the indicated Pi concentration. Pi solubilization index was calculated as described in the Materials and Methods section. Results represent the mean ± SD of three independent experiments. For each panel, different letters indicate significant differences between conditions according to Tukey’s test with a *p* < 0.05.

Several genes involved in phosphorus metabolism were found in the *H. seropedicae* SmR1 genome (NC_014323, Pedrosa et al., 2011), corresponding to approximately 70% of the phosphorus related genes that were identified in the metagenomics approach of soils microbiome performed by Dai et al. (2020). Figure 3A shows that high Pi concentration in culture media unusually increased the expression of some Pi-starvation response components: the Pi regulon two-component response regulator (*phoB*), the AP gene (*phoA*), two Pi starvation-inducible genes (*phoH* and *psiF*), the low-affinity Pi transporter gene (*pitA*), and another Pi transporter encoded by the alkylphosphonate uptake gene (*phnA*). It is noted that, with regard to polyP-related genes, only *ppk2* was induced under the high Pi condition. Regulation of genes involved in polyP synthesis and degradation is complex. It was previously described that fluctuations in polymer levels were not regulated at a genetic level, but by modulation of their enzymatic activity (Rudat et al., 2018).

To analyze whether the observed *phoA* upregulation was correlated with the protein activation, the AP activity was measured in cells grown in NFb5 and in NFb50 media at 4, 9, and 24 h (Figure 3B). A low AP activity was observed when cells grown in NFb5, while, in NFb50, an increased activity was obtained from 9 h. In several bacteria, *phoA* gene transcription is positively regulated only by PhoB, being largely used the AP activity as a reporter of PhoB activation (Wanner and Latterell, 1980; Lamarche et al., 2005, 2008). Interestingly, cells cultivated in NFb50 medium (high polyP condition) overexpressed Pi metabolism genes as if they were sensing Pi deficiency. Previous studies in *E. coli* demonstrated that the maintenance of high polyP levels in stationary phase induced an unexpected activation of PhoB, *via* acetyl phosphate (AcP), in high Pi conditions (Grillo-Puertas et al., 2016). In agreement, when AcP metabolism genes were analyzed in *H. seropedicae* SmR1, an upregulation of the phosphotransacetylase and acetate kinase genes, encoded by *pta* [1.77-fold (*p*-value 0.001)] and *ackA* (3.06-fold), was observed, inferring a possible role of AcP in PhoB activation in *H. seropedicae* SmR1. Considering the scarce previous knowledge about regulation of the Pi genes in *H. seropedicae*, the induction of the classical Pi starvation response in high Pi medium is intriguing. This is in agreement with previous results in *E. coli* (Grillo-Puertas et al., 2016). In addition, it was found that *H. seropedicae* SmR1 was able to solubilize tricalcium phosphate only when 50 mM Pi was added to NBRIP plates (Figure 3C). Although tricalcium phosphate is inappropriate as a universal selection factor for testing Pi-solubilizing bacteria (Bashan et al., 2013), the solubilization of insoluble phosphorus observed here is surprising, not only because previously studied *H. seropedicae* strains did not have phosphorus-solubilizing capacity (Estrada et al., 2013; Wagh et al., 2014), but also due to the observed phenomenon in cells surrounded by excess of environmental Pi. Bagyaraj et al. (2000) suggested the existence of shared regulatory controls between the *pho* regulon and the phosphorus solubilization in Gram-negative rhizobacteria. Phosphorus can be released from organic compounds in soil by different mechanisms, such as the secretion of gluconic acid synthesized by a pathway involving pyrroloquinoline quinone (PQQ)-dependent periplasmic glucose dehydrogenase or the secretion of non-specific phosphatases (Tarafdar and Jung, 1987; Tarafdar and Claassen, 1988; Rossolini et al., 1998). *H. seropedicae* SmR1 is deficient in PQQ biosynthesis genes (Pedrosa et al., 2011); therefore, the aforementioned slight Pi solubilization (Figure 3C) could involve the expression of another organic acid since RNA-seq analysis showed an upregulation of most of the TCA cycle-related genes (*aceE*, *adhA*, *lpdA*, *prpC*, *icd*, *fumA*, *mqo*, and *maeB*) in NFb50 medium (see Supplementary Table 1) and/or the activation of non-specific phosphatases, still uncharacterized in this bacterium. Based on our data, it could be inferred that *H. seropedicae* senses a Pi-deficiency state generated in NFb50 medium, where polyP was not degraded. Indeed, several authors suggested that polyP participates in the balance of the intracellular Pi (Nesmeyanova, 2000; Motomura et al., 2011; Grillo-Puertas et al., 2016).

### Motility and Chemotaxis

**FIGURE 4:**
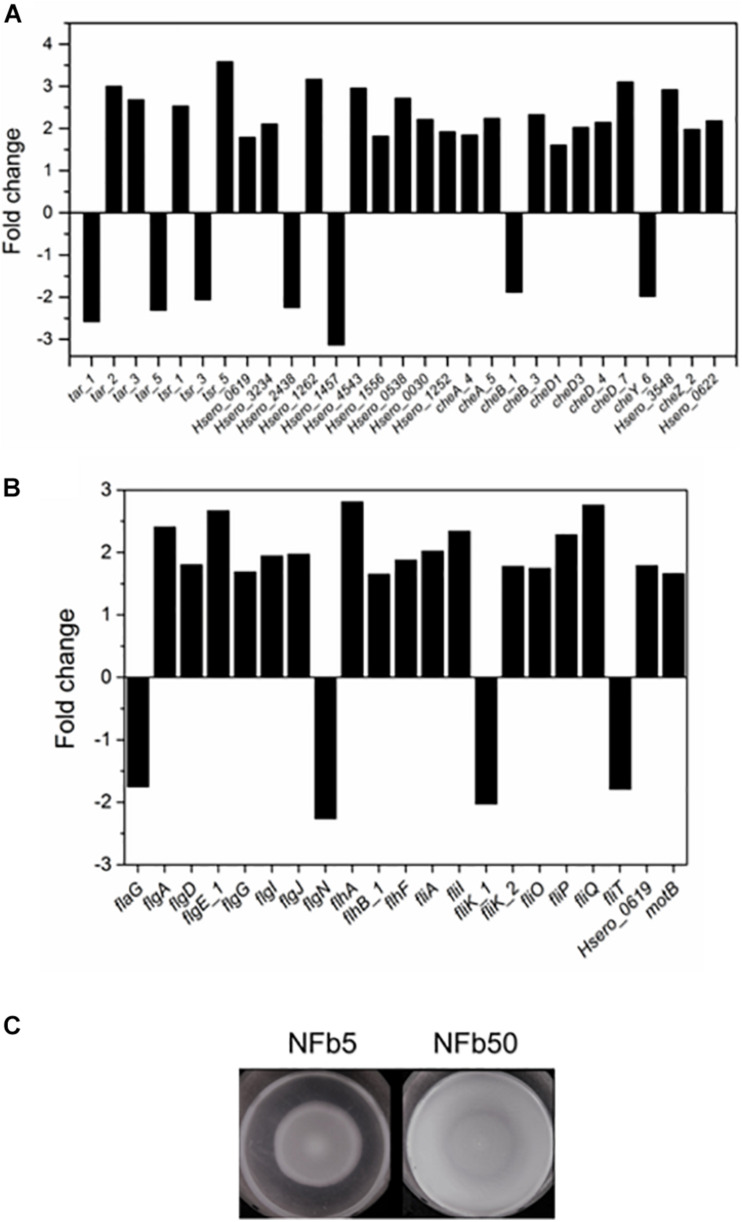
Chemotaxis and motility in NFb50 condition relative to NFb5 condition. Fold changes of differentially expressed genes related to chemotaxis **(A)** and motility **(B)**, comparing both conditions. SmR1 strain cells were cultured on semisolid NFb5 and NFb50 agar plates (0.3%) and incubated for 48 h **(C)**. In all cases, the results represent four independent experiments performed in triplicate.

*H. seropedicae* SmR1 has 41 genes involved in the chemotaxis pathway in its genome (Pedrosa et al., 2011). Figure 4A shows that 22 of 29 motility and chemotaxis DEGs (*tar*, *tsr*, and *che* genes) were upregulated in the NFb50 medium. A wide range (17 from 21 DEG) of flagella biosynthesis, assembly, and structure genes were also induced under the mentioned condition (Figure 4B). For instance, *fliA* gene, encoding a sigma factor necessary for the transcription of flagella and chemotaxis genes (Iriarte et al., 1995; Aizawa, 2013), was significantly induced (2.02). Upregulation of flagella and chemotaxis genes suggests that high Pi concentrations would increase motility and chemotaxis in *H. seropedicae*. Indeed, Figure 4C shows that SmR1 cells grown in NFb50 medium presented a significantly higher motility than those grown in NFb5. Moreover, the cell taxis to carbon sources (malate and glucose), root exudates, and phytohormones (salicylic and shikimic acids) was significantly higher in NFb50 medium than in NFb5 (Table 4). These results suggest that high polyP levels reached in the NFb50 condition could regulate motility in *H. seropedicae*. Consistently, Grillo-Puertas et al. (2015) reported that the presence of polyP is necessary for motility in uropathogenic *E. coli* isolated from acute prostatitis. In addition, previous studies with polyP-deficient strains demonstrated that the polymer is required for motility of several bacteria such as *P. aeruginosa* PAO1, *V. cholerae* 92A1552, *Salmonella enterica* serovar Typhimurium FIRN, *Mycobacterium smegmatis*, and Sulfolobales (Rashid et al., 2000; Shi et al., 2011; Recalde et al., 2021). Chemotaxis and motility differential effects observed with *H. seropedicae* gain attention considering their importance in the properties of beneficial soil microorganisms.

**TABLE 4:** Chemotaxis of SmR1 cells grown in sufficient and high Pi medium.

	NFb5	NFb50
Salicylic acid	0.10 ± 0.03^a^	0.35 ± 0.14^b^
Shikimic acid	0.25 ± 0.05^a^	0.50 ± 0.07^b^
Malate	0.35 ± 0.07^a^	0.40 ± 0.03^a^
Glucose	0.45 ± 0.10^a^	0.65 ± 0.05^b^
Root exudate	0.10 ± 0.0^a^	0.40 ± 0.14^b^
Control	0.20 ± 0.03^a^	0.20 ± 0.02^a^

**Distance of the bacterial growth (cm) in the direction to the attractant in saline solution plates without carbon source containing 0.3% of agar, after 72 h at 30°C. Control corresponds to the same Pi conditions without attractants. Different letters for each attractant indicate significant differences between conditions according to Tukey’s test with a p < 0.05.**

### Biofilm Formation and Adhesion

Genes related to biofilm formation and adhesion were downregulated in cells grown in NFb50 medium: type IV pili genes [*pilI*, *pilJ*, and *pilQ*, with fold change values of −8.71, −1.81 (*p*-value 0.05), and −1.83 (*p*-value 0.003), respectively], the entire cluster for lipopolysaccharide [LPS biosynthesis, comprising CDS *Hsero_4197* to *Hsero_4222* (see Supplementary Table 1)]; and a gene encoding a sugar phosphate isomerase involved in capsule formation, *Hsero_3968* (−2.59). On the other hand, *phoB* was upregulated under the mentioned condition. It was previously demonstrated that PhoB activation was correlated with the inhibition of biofilm formation in *P. fluorescens*, *P. aureofaciens*, and *E. coli* (Monds et al., 2001, 2007; Grillo-Puertas et al., 2016). Thus, the obtained molecular changes suggest that biofilm formation would be impaired in the high Pi condition. To corroborate this hypothesis, biofilm formation was determined in NFb50 and NFb5 media. As expected, it was observed that biofilm formation on abiotic surfaces at 48 h by cells grown in NFb50 medium was around five-fold lower than that of cells grown in NFb5 (Supplementary Figure 1). The fact that biofilm formation was induced in NFb5 medium, where polyP was degraded, suggests that this event is necessary to trigger the cellular adhesion phenotype, as previously observed in K-12 and uropathogenic *E. coli* strains and *G. diazotrophicus* (Grillo-Puertas et al., 2012, 2015, 2018).

**FIGURE 5:**
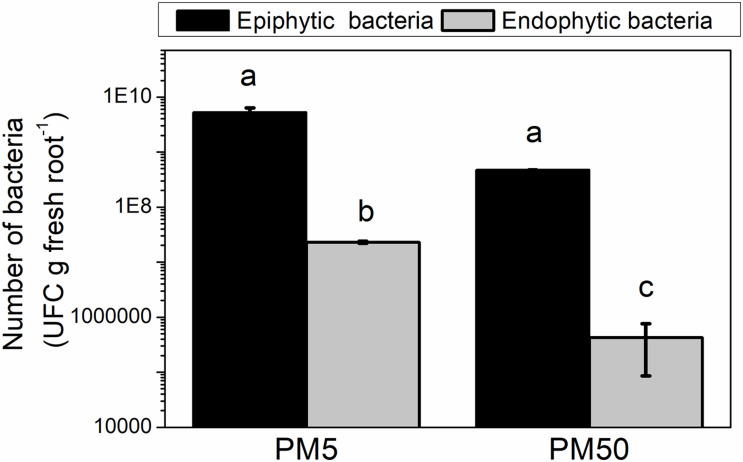
*H. seropedicae* colonization of maize seedlings in the high and sufficient Pi conditions. *H. seropedicae* SmR1 cells were grown in NFb50 medium for 24 h, washed, and inoculated on maize plantlets in PM5 or PM50 (10 replicates per condition). The number of epiphytic and endophytic bacteria was determined after 5 days of infection as mentioned in Materials and Methods. Results represent the mean ± SD of three independent experiments. Different letters indicate significant differences between conditions according to Tukey’s test with a *p* < 0.05.

Besides the genes mentioned above involved in adhesion, *rfbD* and *galE* genes, which have been suggested to be involved in maize colonization (Balsanelli et al., 2010), were downregulated in NFb50 medium (−3.00 and −2.66, respectively). Thus, a plant–bacteria interaction assay was performed to compare the epiphytic and endophytic association capacity of *H. seropedicae* cells to maize roots in plant medium containing either 5 or 50 mM Pi (PM5 or PM50, respectively). Figure 5 shows that epiphytic cell population in the maize root surface was higher in PM5 in respect to PM50, in agreement with data obtained in abiotic adhesion assays. Endophytic population after 5 days of inoculation was approximately 1.5 orders of magnitude higher in cells grown in PM5 when compared to those grown in PM50. It should be noted that the number of planktonic cells was significantly lower in the PM5 medium than that in the PM50 (10^8^ and 10^10^ UFC ml^–1^, respectively), suggesting that the microorganism prefers a sessile lifestyle when grown in the sufficient Pi condition. Taken together, in the sufficient Pi condition (5 mM Pi), *H. seropedicae* cells activate the molecular machinery necessary to improve bacterial adherence to surfaces and root colonization. This is an interesting finding and represents an important outcome to consider in plant–bacteria interaction assays.

### Polyhydroxybutyrate Metabolism

Polyhydroxyalkanoates (PHAs) are a group of carbon and energy storage compounds accumulated by many bacteria to increase survival and stress tolerance in changing environments. Among PHAs, polyhydroxybutyrate (PHB) is a biodegradable polymer used as a possible substitute for petroleum-based plastics (Suriyamongkol et al., 2007; Li et al., 2016). It is well known that polyP form complexes with PHB, generating ion channels in lipid bilayers with high selectivity for cations (Lundgren et al., 1965; Reusch et al., 1995; Das et al., 1997; Pavlov et al., 2005b; Yilmaz and Beyatli, 2005; Zakharian and Reusch, 2007; Reusch, 2012). It has been reported that *phbABC* and *phaC1* genes are responsible for PHB synthesis in *H. seropedicae* SmR1 (Catalan et al., 2007; Kadowaki et al., 2011; Tirapelle et al., 2013). Here, RNA-seq data related to PHB biosynthesis show that *phbA2* and *phbA* genes, encoding two acetyl-CoA acyl transferases, and *phbB* gene, encoding a NADPH-dependent acetoacetyl-CoA reductase, were upregulated in cells grown in NFb50 medium [1.84 (*p*-value 0.0003), 2.5, and 1.51 (*p*-value 0.01), respectively] (Supplementary Table 1). In addition, *phaC1* and *phaC2*, encoding putative PHB synthases, were increased 1.86-fold (*p*-value 0.0001) and 2.18-fold, respectively. The *phaC2* is located in the operon with the *pta-ackA* genes, involved in AcP synthesis (Pedrosa et al., 2011). In cyanobacteria, AcP seems to play a role controlling PHA synthase (Sundaramoorthy et al., 2013). In the studied conditions, the co-expression of the *pta-ackA* with *phaC2* genes suggests the participation of AcP in PHB metabolism in *H. seropedicae*.

**FIGURE 6:**
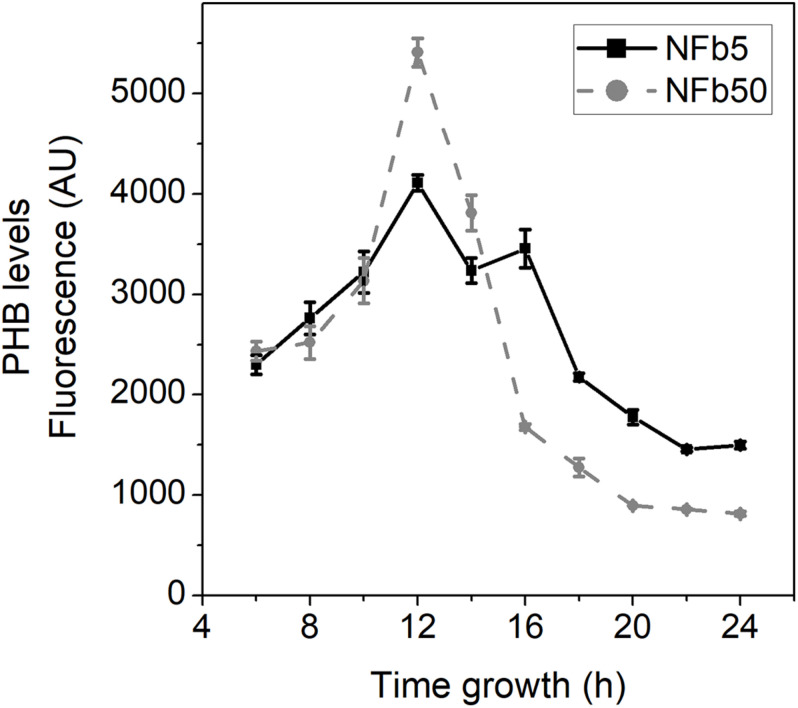
PHB levels in SmR1 cells grown in NFb50 and NFb5 condition. PHB measurements by flow cytometry in Δ*phaC1* and SmR1 strains cultivated in NFb5 and NFb50 media. At the indicated times, culture aliquots were extracted, stained with Nile Red, and analyzed by flow cytometry as mentioned in the Materials and Methods section. Data are expressed as arbitrary units (AU) of fluorescence. Results represent the mean ± SD of three independent experiments with a *p* < 0.05.

To analyze the physiological response to the aforementioned genes’ differential expression, PHB levels were measured through time during *H. seropedicae* SmR1 growth in NFb5 and NFb50 (Figure 6). In both media, PHB levels were increased up to 12 h, and thereafter decreased. It should be noted that the highest values of PHB were reached in NFb50, in agreement with the RNA-seq data. In *Azospirillum brasilense*, accumulation of PHA has been related to chemotaxis, motility, and cell multiplication, becoming a more efficient inoculant (Kadouri et al., 2005; Fibach-Paldi et al., 2012). In fact, it has been described that PHB increased plant growth promotion in *H. seropedicae* and *A. brasilense* (Kadouri et al., 2003; Alves et al., 2019). However, previous studies with *H. seropedicae* SmR1 and *A. brasilense* Sp7 found no significant differences in epiphytic or endophytic root colonization, when inoculated with wild-type strains or their PHB-deficient mutants (Kadouri et al., 2003; Balsanelli et al., 2016; Alves et al., 2019). Even though a higher PHB peak was observed in NFb50 cells at 12 h, the synthesis and degradation profiles of the polymer were similar in both conditions. Further studies would be necessary to shed light on the possible relationship between PHB and the observed differential phenotypes. Moreover, the possibility of increasing polymer production in a given time and condition would represent a satisfactory approach for the production of environmentally friendly plastics.

### Other Traits Affected by Pi

Nitrogen metabolism and its regulation have been extensively studied in *H. seropedicae* SmR1 (Chubatsu et al., 2012). Nitrogen assimilation in this bacterium is regulated by the NTR system, which is responsible for activating transcription of genes involved in alternative nitrogen sources in nitrogen-depleted conditions (Persuhn et al., 2000; Wassem et al., 2002; Schwab et al., 2007; Chubatsu et al., 2012). Supplementary Table 1 shows that *H. seropedicae* cells grown in NFb50 medium induced some of the NTR system genes such as *ntrC* (2.63), *ntrB* [1.99 (*p*-value 0.0006)], and *glnA* [1.63 (*p*-value 0.01)] and the operon *amtBglnK* [2.24 and 1.54 (*p*-value 0.006)]. Also, the gene involved in ammonium uptake *amtH* (4.60) was induced. Considering that, in the present work, cells were grown in sufficient nitrogen conditions, differential expression of these genes could be in response to unknown mechanisms that may be related to functions other than nitrogen/nitrate metabolism in the high Pi condition. For instance, it has been reported that an increase in AcP could activate NtrC, inducing Ntr regulon in several bacteria (Feng et al., 1992; Verhamme et al., 2002), since, in certain conditions, this molecule is sufficient for direct phosphorylation of two-component response regulators (Kim et al., 1996; Klein et al., 2007).

ATP-binding cassette (ABC) transporters catalyze the translocation of various substrates across the bacterial membrane. They enable the uptake of nutrients and important molecules and facilitate the extrusion of toxins (Dawson and Locher, 2006). The RNA-seq analysis showed a significant regulation of ABC-transporter genes (42 DEGs) in response to Pi concentration, with 16 being downregulated and 26 being upregulated in NFb50 medium (Supplementary Table 1). Among the downregulated genes, the most relevant were those involved in carbohydrate and ion transport (i.e., glycerol-3-phosphate, Fe^3+^, and sulfate/molybdate transporters). On the other hand, among the upregulated ABC transporters genes, the operon *tauABC*, responsible for taurine (aliphatic organosulfonate) transport inside the cell for further degradation into alanine, was significantly induced (fold change 4.5–10). The importance of this sulfonate transport system has been described for several bacterial species, such as *S.* Typhimurium (Turnbull and Surette, 2010), *P. aeruginosa* (Hummerjohann et al., 1998), *Bacillus subtilis* (Grundy and Henkin, 1998), and *Acidithiobacillus ferrooxidans* (Valdés et al., 2003). In addition, sulfated metabolites have been implicated in the interactions between bacteria and their eukaryotic hosts, including species of the plant symbiont genus Rhizobium (Cronan and Keating, 2002), *Mycobacterium tuberculosis* (Mougous et al., 2002), and *Xanthomonas oryzae* (Da Silva et al., 2004). Several DEGs predicted for amino acid transport and metabolism were upregulated in NFb50 medium (*livH_4*, *livK_5*, *livK_3*, *livF_1*, *Hsero_4792*, *livK_1*, and *hisJ_1*), together with the ABC transport genes for putrescine (*potCHDA*, *Hsero_1078-1079-1080-1081*, and *Hsero_0600*). Putrescine or 1,4-diaminobutane is a biogenic polyamine present in nearly all living cells and is involved in bacterial response to osmotic stress (Miller and Wood, 1996). The rapid bacterial adaptation to the osmolarity would facilitate the colonization of the rhizosphere (Miller and Wood, 1996). Also, the amount of this compound in plant cells increases in some stress situations (Flores and Galston, 1982). Therefore, putrescine transporters could also be involved in the bacterial protection from the putrescine produced by the colonized plant. Interestingly, three multidrug ABC transporter genes (*mdlB*, *Hsero_4073*, and *Hsero_3566*) were also upregulated by 1.60 (*p-value* 0.03), 1.57 (*p-value* 0.008), and 1.58 (*p-value* 0.03), respectively. Multidrug transporters mediate the extrusion of structurally unrelated drugs from prokaryotic and eukaryotic cells (Blackmore et al., 2001). Upregulation of these transporters would be important to *H. seropedicae* defense against different compounds present in the rhizosphere. According to the observed robust response of ABC transporter systems, it can be suggested that Pi is an important signal regulating bacterial transport processes in response to environmental conditions.

Central metabolism requires a fine regulation to ensure optimal use of carbon sources and proper allocation of resources to the metabolic pathways (Neidhardt et al., 1990; Heinrich and Schuster, 1998; Karp et al., 2007). It has been reported that polyP acts as a buffer for free Pi level (Kornberg et al., 1999). Since Pi buffering is essential for general metabolism, the synthesis or degradation of the polymer has been associated with the regulation of primary metabolism and general fitness in prokaryotic and eukaryotic cells (Freimoser et al., 2006; Schurig-Briccio et al., 2008, 2009a,b; Varela et al., 2010; Livermore et al., 2016). Within carbon metabolism, the tricarboxylic acid (TCA) cycle is an amphibolic pathway involved in energy production and precursor biosynthesis in aerobic organisms (Sauer and Eikmanns, 2005). Through its anabolic function, the TCA cycle produces 2-oxoglutarate and oxaloacetate as precursors of the glutamate family (glutamate, glutamine, arginine, and proline) and the aspartate family (aspartate and asparagine) of amino acids, respectively (Bott, 2007). Before it enters the TCA cycle, acetyl-CoA is generated by the pyruvate dehydrogenase complex (PDC) (Patel and Roche, 1990). Here, genes encoding for the pyruvate dehydrogenase components (*aceE1*, *lpdA*_1, *lpdA_2*, and *aceF*) were upregulated in NFb50 growing cells [6.69, 2.55, 2.00, and 1.56 (*p*-value 0.004), respectively]. In addition, most of the genes encoding for TCA cycle enzymes were upregulated in the high Pi condition, such as *prpC* (11.01), *acnA_1* [1.46 (*p*-value 0.02)], *acnA_2* [1.66 (*p*-value 0.003)], *icd_2* (2.57), *sucB* [1.68 (*p*-value 0.001)], *lpdA_1* (2.55), *lpdA_2* (2.00), *sucD* [1.77 (*p*-value 0.001)], *fumA* (2.09), *and mqo* (4.59). No significant DEGs related to the oxidative phosphorylation pathway were found, suggesting that the upregulation of the TCA cycle genes in the high Pi condition would supply intermediates for biosynthetic pathways. Actually, several genes related to amino acid metabolism were upregulated in the aforementioned condition. For instance, *ansB_1*, *argJ*, *argE, argH*, *Hsero_0061*, *Hsero_4778*, and *Hsero_4377* genes, involved in arginine and proline metabolism; *ansB_1*, *ansB_2, gltB, gltD*, and *goaG* genes, involved in alanine, aspartate and glutamate metabolism; *thrC* and *thrA* genes belonging to threonine metabolism; and *dadA*, *Hsero_0324, Hsero_1676, Hsero_1447, Hsero_0324, Hsero_1684, Hsero_2240*, and *Hsero_4778* from phenylalanine metabolism (see Supplementary Table 1). In particular, *Hsero_4778* gene, described to be essential for *in vitro* growth of *H. seropedicae* (Rosconi et al., 2016), encoded for an enzyme that catalyzes the interconversion of pyruvate and D-glutamate to D-alanine and 2-oxoglutarate, providing a carbon skeleton for the TCA cycle. Moreover, the TCA cycle shares common intermediates with the glyoxylate cycle, with both pathways being coordinately regulated. The glyoxylate shunt is known to be upregulated when acetyl-CoA is a direct product of a metabolic pathway, for example, *via* degradation of acetate, fatty acids, and alkanes (Maloy and Nunn, 1982; Chung et al., 1993). Note that *glcD*, *glcE*, and *aceA* genes, coding for the specific enzymes of the glyoxylate cycle, were downregulated in the high Pi condition (−5.42, −5.39, and −7.60, respectively). There is evidence suggesting that the glyoxylate shunt also plays an important role in pathogenesis and the response to oxidative stress in microorganisms (Ahn et al., 2016). Although it is not possible to predict metabolic fluxes from transcriptomic data, these results highlight the fact that environmental Pi is capable of modulating the expression of most of the genes from certain central metabolism pathways in *H. seropedicae.*

Many bacteria respond to oxidative stress by changing the expression of genes encoding enzymes that regulate the cellular concentration of superoxide and hydrogen peroxide, the formation of iron–sulfur clusters, and the reparation of oxidative damage to DNA (Imlay, 2008; Gray and Jakob, 2015). Here, different stress global regulators, such as *soxR* (-4.91), *norR* (-4.55), *acrR* (-3.10), *ompR* (-2.67), and *risA* (-2.06); two genes involved in the SOS response for DNA repair, *recX* (-2.61) and *umuD* (-2.21); and a gene encoding a superoxide dismutase protein, *sodB* (-1.98), were downregulated in NFb50 medium (Supplementary Table 1). Also, around 11 LysR-type transcriptional regulator (LTTR) genes were significantly downregulated in the same medium (*Hsero_3928, Hsero_4799, Hsero_3540, cysB, Hsero_2318, Hsero_4110, Hsero_0047, Hsero_4802, Hsero_4528, Hsero_3808*, and *Hsero_4346*). LTTRs are a well-characterized group of transcriptional regulators that control key adaptive phenotypes, such as virulence, biofilm formation, quorum sensing, motility, signaling, secondary metabolite production, and oxidative stress (Russell et al., 2004; Tropel and van der Meer, 2004; Heroven and Dersch, 2006; Maddocks and Oyston, 2008; O’Grady et al., 2011). In addition, genes encoding different bacterial chaperones, such as *ibpA*, *groES*, *htpX*, *Hsero_1726*, *Hsero_0435*, and *hfj*, were also significantly downregulated in NFb50 (Supplementary Table 1). Our results indicate that an important stress response is observed only in cells grown in NFb5 medium (low polyP condition), suggesting that cells sense and respond to the usual deprived state of the early stationary phase. It was widely reported that polyP protects bacteria from cellular stresses (Jahid et al., 2006; Rao et al., 2009; Nikel et al., 2013; Alcántara et al., 2014; Gray et al., 2014). PolyP can act directly by a protein-stabilizing chaperone activity and by interactions with redox-active metals, or indirectly controlling stress response pathways (Gray and Jakob, 2015). Previous reports from our laboratory indicate that high polyP levels in stationary phase improve bacterial fitness and resistance to different stresses (Schurig-Briccio et al., 2009a; Grillo-Puertas et al., 2014, 2018). Thus, the downregulation in the NFb50 condition of these stress-related components may be due to the chaperone capacity of the polyP that could provide the cytoplasmic protein quality to overcome a stress condition or due to the fact that cells grown in high Pi medium do not sense the typically early stationary phase-stress condition (Zhaoa and Drlica, 2014).

## Conclusion

**FIGURE 7:**
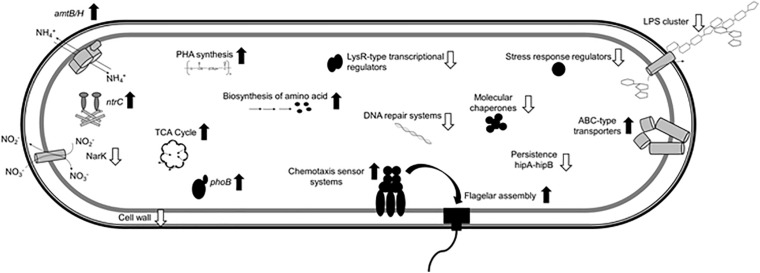
Molecular differences of *H. seropedicae* cells in the high Pi condition relative to the sufficient Pi condition. The scheme shows induced (black up arrows) and repressed (white down arrows) traits in *H. seropedicae* SmR1.

The present study provides a comprehensive analysis of the *H. seropedicae* SmR1 transcriptome in Pi differential conditions. Figure 7 shows a general scheme of some of the molecular adaptations that occurred in cells grown in medium containing 50 mM Pi when compared to those grown in 5 mM Pi. Transcriptional analysis revealed that 670 genes were differentially expressed, with 57% repressed (i.e., adhesion and stress response) and 43% induced (i.e., Pi metabolism, bacterial flagella biosynthesis, chemotaxis, energy production processes, and PHB metabolism) in the high Pi condition. Based on transcriptomic and phenotypic data, *H. seropedicae* cells grown in high Pi medium seem to have a better general fitness, which could be an important feature to consider when the bacterium is used as plant inoculant. On the other hand, under sufficient Pi concentrations, cell adhesion and colonization capacity increased, leading to a higher root colonization. The present data shed light on the bacterial behavior in respect to changes in environmental Pi concentrations that could be useful for the development of strategies to benefit their biotechnological and agricultural potential.

## Data Availability Statement

The datasets presented in this study can be found in online repositories. The names of the repository/repositories and accession number(s) can be found below: https://www.ncbi.nlm.nih.gov/geo/, GSE168341.

## Author Contributions

MG-P, ES, and VAR conceived and designed research. MG-P, JMV, VP, MT-S, EH, and FT conducted experiments. LB-S contributed to DESeq analysis. RP contributed to general ideas and formulation of the research aims. MG-P, JV, and VAR analyzed the data. FP, ES, and VAR provided financial support. MG-P, JMV, VAR, and ES wrote the manuscript. All authors read, discussed, and approved the manuscript.

## Conflict of Interest

The authors declare that the research was conducted in the absence of any commercial or financial relationships that could be construed as a potential conflict of interest.

## Footnotes

1 https://www.ncbi.nlm.nih.gov/geo/
