# Supplementary material for: Transcriptional Responses of Herbaspirillum seropedicae to Environmental Phosphate Concentration
Source: Front Microbiol. 2021 Jun 10;12:666277. doi: 10.3389/fmicb.2021.666277 (PMC8222739; doi:10.3389/fmicb.2021.666277)
Supplement: Supplementary file 1 [file Data_Sheet_1.PDF]

1.1 Supplementary Figure 1

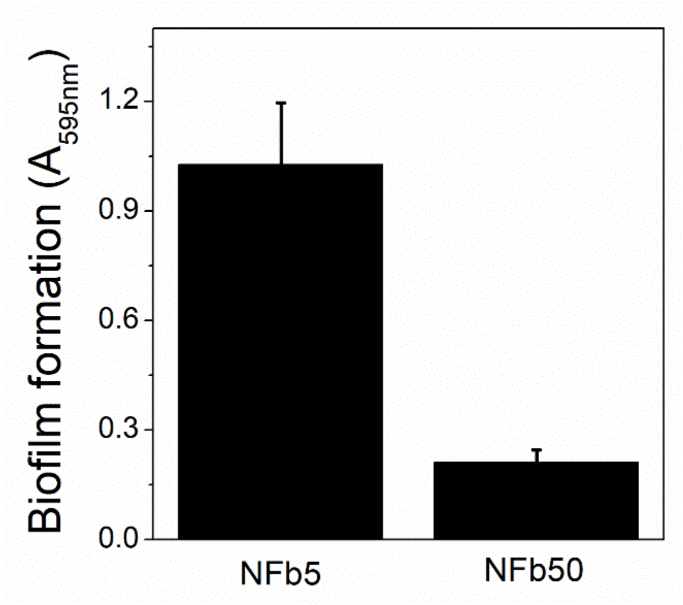

Supplementary Figure 1. *H. seropedicae* SmR1 cells were grown in static conditions during 48 h at 30°C in the indicated media. The biofilm amount was determined by the crystal violet assay as mentioned in Experimental procedures
